# Efficacy and Challenges in the Treatment of Diastasis Recti Abdominis—A Scoping Review on the Current Trends and Future Perspectives

**DOI:** 10.3390/diagnostics12092044

**Published:** 2022-08-24

**Authors:** Menaka Radhakrishnan, Karthik Ramamurthy

**Affiliations:** Centre for Cyber Physical Systems, School of Electronics Engineering, Vellore Institute of Technology, Chennai 600127, India

**Keywords:** DRA, physiotherapy, EMG, rehabilitation

## Abstract

Diastasis recti abdominis (DRA) is more prevalent in women during pregnancy and postpartum. However, there is a lack of awareness regarding this condition among women. The prevalence of DRA is high in late pregnancy and reduces during postpartum. The purpose of this study is to provide an overview of the treatment strategies for DRA and to discuss the significance of the technology towards better diagnosis and treatment. This work investigated 77 research articles published in the recognized research databases. The study aims to analyze the diagnostic and treatment procedures and the role of technology within them. The management strategy for DRA can either be conservative or surgical. Exercise therapy has been shown to improve functional impairments. These exercises focus on recruiting the abdominal muscles. Electromyography and Ultrasound imaging have been employed as useful tools in assessing the abdominal muscles effectively. This study has examined the treatment methods for DRA to obtain a better understanding of the existing methods. Further investigation and experimentation into therapeutic exercises is strongly recommended to identify the best set of exercises for a faster resolution. Further studies regarding the role of technology to assess therapeutic exercises would be worthwhile.

## 1. Introduction

Pregnancy and postpartum affect the female body in numerous ways. During this period, women’s bodies tend to undergo hormonal, anatomical, and physiological variations. While these changes provide a feasible environment for the development of the foetus, they also impose a negative influence on the musculoskeletal health of the female body. As a result, women experience unusual musculoskeletal issues in the abdomen after childbirth. One such issue is DRA [1]. It has received much attention in the last two decades among researchers and women. It is a benign condition characterized by the separation of the rectus abdominis muscles [2]. The main problem with much of the literature regarding the prevalence of DRA is that it varies according to the sample size, methods used to measure the IRD, the cut-off value of IRD, and the inclusion and exclusion criteria of patient selection. Bo et al. performed a prospective cohort study of primiparous women during pregnancy and postpartum. A prevalence rate of 33.1%, 60%, 45.4%, and 32.6% at gestational week 21, 6 weeks postpartum, 6 months postpartum, and 12 months postpartum, respectively, were reported in this study [3]. In a longitudinal observational study, Mota et al. investigated the prevalence of DRA in primiparous women from the 35th gestational week, and 6–8, 12–14, and 24–26 weeks postpartum. They found that the prevalence of DRA decreased from 100% at gestational week 35 to 39% at 6 months postpartum [4]. In a recent study, Fei et al. reported 82.6% as the prevalence rate in their retrospective study conducted among women in their first year of postpartum [5].

Risk factors for DRA do not appear to be well-grounded and need to be examined in greater depth. Pregnancy, cesarean section, maternal age, multiparity, foetal macrosomia, multiple gestations, age, obesity, the performance of full-excursion sit-ups, weight training, abdominal wall stress, and ethnicity are some of the risk factors reported by the several studies in the literature. However, the association of each risk factor with DRA requires further investigation. [6,7,8]. Researchers have reported numerous findings regarding the consequences of DRA. Changes in abdominal muscle function, pelvic floor muscle dysfunction, and pain in the lower parts of the body are the physical symptoms in women at different stages after delivery. In a few cases, DRA may be negatively associated with the strength of the rectus abdominis and oblique muscles [9]. However, the experience of abdominal pain is positively associated with DRA in women during their early postpartum period. However, its intensity seemed to be low and clinically irrelevant [10,11]. Pelvic floor dysfunctions include urinary incontinence, fecal incontinence, and pelvic organ prolapse. It is reasonable for these to be present in peri- and postmenopausal women and urogynaecology patients [12,13]. Low back pain and pelvic girdle pain are the other consequences investigated in the literature. A study concluded that low back pain may be related to DRA [14]. DRA affects women in multiple ways. Erikkson et al. conducted a study which reflected women’s experiences living with increased IRD after childbirth. Women in this study reported diminishing changes in their body image, function, and ability than before. They also mentioned that there was a lack of awareness of DRA among them [15].

DRA is diagnosed by measuring the distance between the two recti muscles. Abdominal palpation, tape measures, calipers, and ultrasound are the most common methods used in clinical practice. Clinicians also use imaging techniques, such as computer tomography (CT) and magnetic resonance imaging (MRI), to measure the distance between the two recti muscles. Such imaging techniques provide accurate results, but they are expensive. CT imaging exposes women to unnecessary radiation. Surgeons use intraoperative tapes, rulers, and compasses for the measurement. The majority of women undergo a natural resolution of diastasis recti during the initial stage of their postpartum period. However, in some cases, DRA persists and tends to cause functional and cosmetic defects in women’s bodies. Therefore, women with visible symptoms of DRA seek medical advice. Conservative therapy is the treatment prescribed initially. However, when conservative therapy seems ineffective in severe cases, women are directed towards surgical intervention. Both treatments aim for improvements in the morphological, functional, and quality of life of patients.

Surgeons approach DRA in many ways. There are open, laparoscopic, endoscopic, and hybrid techniques. Each method has its own benefits and drawbacks. In each method, different procedures are available that surgeons can choose, based on their experience in treating DRA. The patients with symptomatic DRA without any cosmetic defects get appointed to the general surgeon, while the plastic surgeon treats DRA patients with cosmetic disabilities [16]. Intraoperative and postoperative complications include pain, wound infections, hematoma, seromas, mesh extrusions, vasomotor changes, skin burns, nerve injury, pneumonia, minor dehiscence, umbilicus site infection, numbness, or paresthetic sensations in the abdominal skin. Complications arising due to surgery differ with each patient’s operation and with the varying techniques used, according to the literature [17,18,19].

DRA may occur even after the patient undergoes a surgical procedure. Few studies in the literature have reported an insignificant recurrence rate [20,21]. Conservative treatment studies for DRA are scarce. It is considered the primary treatment for DRA. It involves physiotherapeutic exercise training, and abdominal binding. While one group of researchers claim to engage the rectus abdominis muscle, another group of researchers claim to engage the transverse abdominis muscle for effective rehabilitation. Hence the exercise protocols which provide effective results need to be investigated. Although there is a lack of evidence in exercise therapy, the main advantage is that it is a noninvasive treatment. In clinical practices and research, the effectiveness of exercises in DRA patients is assessed by measuring the IRD. This measurement is performed manually by the therapist and is quite a long and tedious process. An automatic assessment method for IRD may be beneficial to both the therapist and patient. It may serve as constant feedback and provide results as and when required to the patients.

This review is organized into the following sections: Section 2 describes the search strategy followed to collect the articles from the databases; Section 3 provides a brief on the condition, DRA, and the measurement methods used to measure the IRD; Section 4 explains the invasive and noninvasive treatment methods used to manage DRA; Section 5 describes the diagnostic techniques in the assessment of abdominal muscles during exercises; Section 6 discusses the technical requirements that would improve the diagnosis and treatment of DRA; Conclusively, Section 7 addresses the importance of the role of technology in the rehabilitation of postpartum women with DRA.

The following are the scope and key objectives of this research:

1. To analyze the different diagnostic and treatment procedures associated with DRA.
2. To analyze the challenges involved in the assessment of treatment and rehabilitation procedures.
3. To highlight the potential research gaps and future trends relating to the treatment of DRA.

## 2. Search Strategy and Organization of the Review

**Figure 1:**
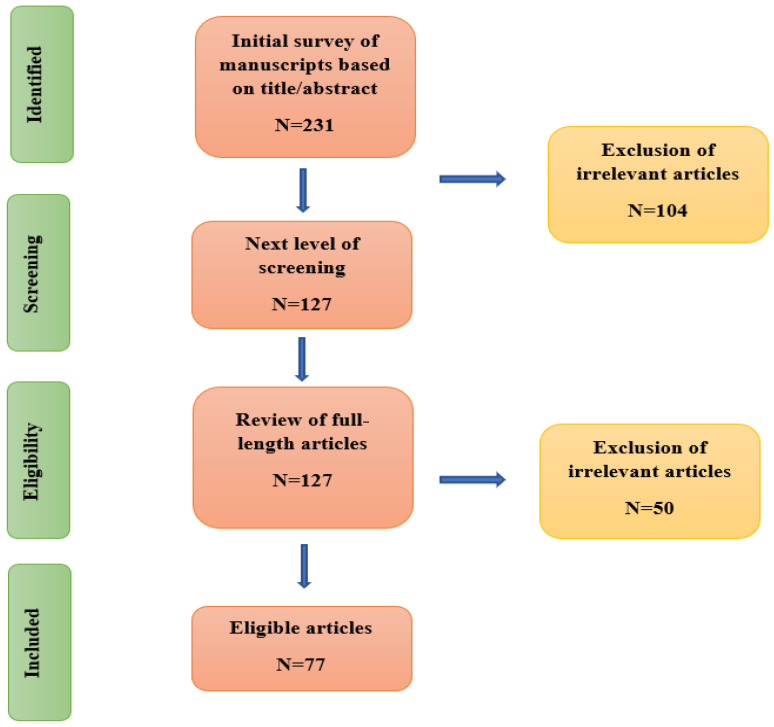
Search strategy and filtering process.

**Figure 2:**
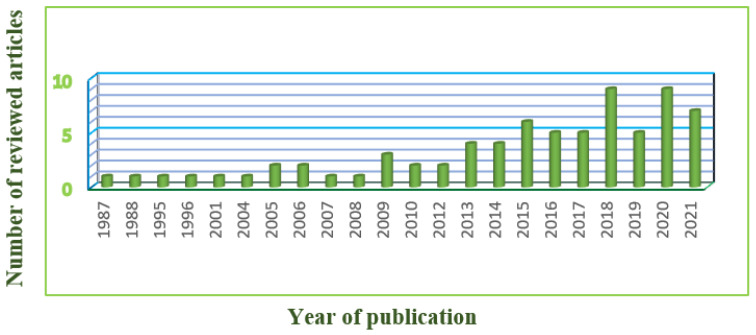
Year-wise distribution of the reviewed articles.

The articles relating to DRA were explored in seven databases, namely PubMed^®,^ ScienceDirect^®^, SpringerLink, PubMed Central^®^, Wiley Online Library, Google Scholar, and Cochrane Library. Suitable published articles were found and downloaded using keyword searches, using terms such as ‘Diastasis Recti Abdominis’, ‘Physiotherapy’, ‘Physical therapy’, ‘exercises’, ‘pregnancy’, ‘postpartum women’, ‘treatment’, ‘linea alba’, ‘prevalence’, ‘rehabilitation’, ‘abdominal exercises’, ‘Electromyography’, and ‘Ultrasound Imaging’. From the forementioned databases, we collected 231 articles, which explains that only very scarce research has been conducted on this topic. After applying filtering techniques, the number of articles was reduced to 77. Figure 1 presents the filtering process applied to finalize the relevant articles. Figure 2 illustrates the year-wise distribution of the published articles relating to DRA. It is inferred that there has been an increase in research on DRA only in the last decade. The inclusion and exclusion criteria of this research work is presented in Table 1.

The inclusion and exclusion criteria for this research work is presented in Table 1. 

## 3. DRA—A Walkthrough

This section provides a brief overview of the anatomy of the rectus abdominis muscle, morphological changes in DRA, the width of the linea alba, and the measurement methods used to measure the IRD.

### 3.1. Anatomy of Rectus Abdominis Muscle

The three layers of the anterior abdominal wall are the skin and adipose tissues, the myofascial layer, and a deep layer. The myofascial layer is the middle layer of the anterior abdominal wall and is composed of muscles and their fascial envelopes. It holds two vertical paramedian muscles and three lateral muscles. 

**Figure 3:**
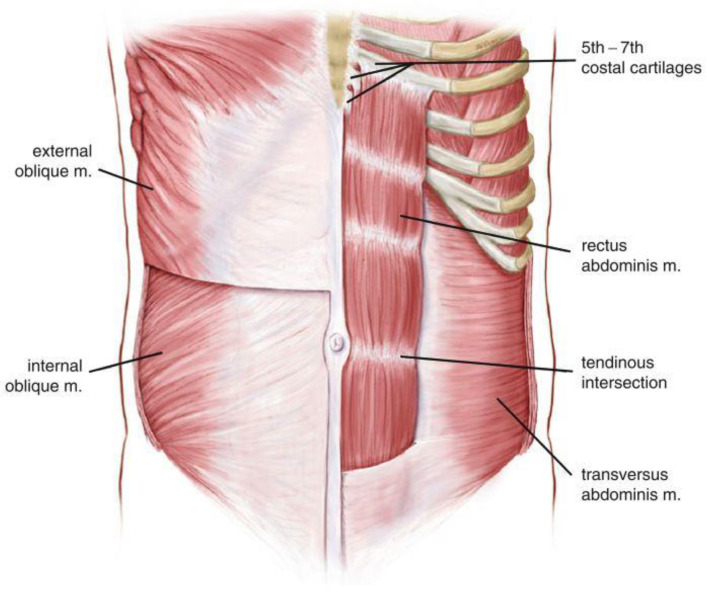
Anatomy of abdominal muscles [2].

The rectus abdominis muscle occupies the central part of the anterior abdominal wall. It is a vertical-oriented paired strap muscle which originates from the pubic symphysis, crest, and pecten. It then runs vertically upwards and is inserted into the xyphoid process and the fifth to seventh costal cartilages. The fibrous structure, linea alba, that runs vertically from the xyphoid process to pubic symphysis, separates the paired rectus abdominis muscles at the midline of the abdomen. A curved depression called the Linea semilunaris forms the lateral borders of the rectus abdominis on either side. Rectus abdominis muscles are also separated horizontally by three to four horizontal tendinous intersections. The three fibrous band intersections are located at the umbilicus, at the level of the xyphoid process, and halfway between these two. The fourth location may be below the umbilicus, which denotes the location of the arcuate line [22,23]. Figure 3 presents the anatomy of the abdominal muscles.

### 3.2. Morphological Changes in DRA

**Figure 4:**
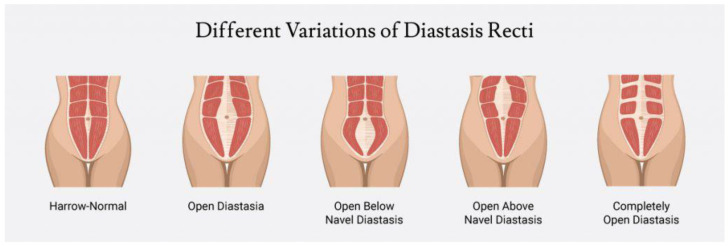
Diastasis Recti at different locations [33].

DRA occurs during the second or third trimester of pregnancy. During pregnancy, women’s bodies tend to undergo several anatomical, physiological and hormonal changes. The growing foetus alters the morphology of the abdomen muscles in pregnant women. Due to hormonal changes affecting the connective tissue, the linea alba extends and the laxity increases. Additionally, the increased intra-abdominal pressure for a prolonged period of time results in the elongation and separation of the rectus abdominis muscle. As a result, the width of the linea alba, or the distance between the two rectus muscles, increases. This condition is often visible as bulging of the midline of the stomach while performing specific movements. It develops above, below, or at the umbilicus, or anywhere along the length of the linea alba [24,25,26]. Figure 4 represents diastasis recti at different locations along the linea alba.

### 3.3. Width of the Linea Alba

The width of the linea alba varies across the regions of the anterior abdominal wall. The width of the linea alba from the xyphoid process to the umbilicus ranges from 11 to 21 mm, and from the umbilicus to the pubic symphysis, the width decreases from 11 to 2 mm [2]. In the literature, two studies evaluated the normal width of the linea alba. A study with 150 nulliparous women by Beer et al. assessed the normal width of the linea alba at three reference locations. They reported a normal linea alba width of up to 15 mm at the xyphoid, 22 mm at 3 cm above the umbilicus, and 16 mm at 2 cm below the umbilicus. Another longitudinal descriptive exploratory study by Mota et al., with 84 primiparous women, at four-time points from pregnancy to 6 months postpartum, revealed that the width varied along the linea alba. At all four time points, the width of the linea alba was widest at 2 cm above the umbilicus [27,28].

### 3.4. Measurement Methods

The measurement of IRD in DRA patients is significant in clinical practices. The measurement is performed to identify the presence of DRA, or to monitor the outcome measures across different treatment options towards DRA. In the literature, researchers and clinicians report several measurement methods. The measurement methods include palpation, assessed by finger width, tape measure, calipers, and ultrasound. CT, MRI, intraoperative rulers, and compass measurement have also been utilized. Abdominal palpation is the most commonly used assessment method in clinical practice. Over the studies performed in the literature, the IRD cut-off value of finger width, greater than or equal to 2 cm, was used as the diagnostic criteria for DRA. However, studies vary with certain criteria, such as measurement location, the position of the patient, and the task performed by the patients during measurement [29,30,31].

Sperstad et al. classified women into four categories based on the largest IRD measured at three locations by palpation. Their classification reports patients with <2 finger width as non-DRA, 2–3 finger width as mild diastasis, 3–4 finger width as moderate diastasis and, >4 finger width as severe diastasis [10]. Studies utilizing calipers as their measurement method have fixed the cut-off value for IRD derived from either Rath et al. or Beer et al., despite the difference in measurement location. Rath et al. expressed DRA in a patient when the IRD is >10 mm above the umbilicus, 27 mm at the umbilical ring, and 9 mm below the umbilicus [8,13]. A recent study conducted by Qu et al. established an ultrasound diagnostic criterion for DRA. The first criterion is an IRD of >2 mm at 3 cm below the umbilicus. The second criterion is an IRD of >20 mm at the umbilicus, and the third criterion is an IRD of >14 mm at 3 cm above the umbilicus. According to these criteria, the authors classified postpartum women into six types of groups. Women who met only the first criterion were classified as the sub-umbilical separation type, the second criterion as the umbilical separation type, the third criterion as the supraumbilical separation type, the first and second criteria as umbilical and sub-umbilical separation types, the second and third criteria as umbilical and supraumbilical separation types. Women who met the criteria at all three locations were classified as complete separation type [32].

## 4. Treatment

The management techniques for DRA can be invasive, minimally invasive, and noninvasive depending on the severity. Surgical techniques are either invasive or minimally invasive. Noninvasive treatments include conservative treatments, such as physiotherapy, visceral manipulation and abdominal binding. This section describes the surgical and conservative treatments prescribed for DRA.

### 4.1. Conservative Treatment

Conservative treatment for DRA includes an abdominal binder, electrical stimulation, manual therapy, and therapeutic exercises. From the available methods, therapists often use exercise and abdominal binders as treatment. Exercise is the most commonly used modality by therapists to approach DRA. Abdominal binders may provide physical support to the abdominal wall when used in the early postpartum period. Recent studies using electrical stimulation and manual therapy methods to treat DRA have also shown promising results.

#### 4.1.1. Non-Exercise Physical Therapy

Not many studies have been conducted to analyze the effect of non-exercise physical therapies on DRA, such as abdominal binders, kinesiotape, electrical stimulation, and manual therapy. El-Mekawy et al. conducted a study to determine the effect of the abdominal supporting belt with abdominal exercises on IRD separation and abdominal efficiency. This study was performed with 30 primiparous postnatal women divided into two equal groups. The first group had 15 women who utilized abdominal belts from the second day until 6 weeks after delivery. The second group had 15 women who performed abdominal exercises 3 times a week from the second day until 6 weeks after delivery. The authors reported that abdominal exercises significantly increased abdominal strength and decreased IRD in women, compared to the use of an abdominal belt [34]. A pilot RCT by Keshwani et al. compared the effect of exercise therapy and/or abdominal binding on 32 primiparous women. These women were divided into three groups undergoing exercise therapy, abdominal binding, and a combination of both therapies. Positive outcomes were witnessed in abdominal binding alone, as well as combinational therapy of both after 6 months [35]. Another pilot RCT by Tuttle et al. analyzed the effectiveness of TrA exercises and kinesiotape on 30 women. The study consisted of four groups, a control group, and three experimental groups who underwent TrA exercises, kinesiotaping, and a combination of both. It was observed that significant changes in IRD were observed in TrA exercise and combination groups only [36].

In recent times, therapists have been utilizing electrical stimulation methods to reduce IRD in DRA patients. Kamel et al. assessed the effect of Neuromuscular Electrical Stimulation (NMES) in postnatal women [37]. They concluded that NMES helps to decrease IRD in postnatal women, and when combined with abdominal exercises, the outcomes may be enhanced. Wang conducted a study on electrical stimulation for DRA and assessed the outcomes using ultrasonography. His outcomes were also similar to the previous study, resulting in a decrease in IRD after treatment [38]. Visceral manipulation (VM) manual therapy was recently first used in the treatment of DRA. Kirk et al. conducted a retrospective chart review of three women diagnosed with DRA. Four treatments of VM were given to all three patients and all of them demonstrated a decrease in IRD [39].

#### 4.1.2. Physical Exercise Therapy

Six to eight weeks after delivery, women with DRA are advised to commence exercises as a conservative therapy. Physiotherapy is considered to be a first-line treatment for DRA. Therapists prescribe effective abdominal exercise during pregnancy and postpartum. The description of a few of the prescribed abdominal exercises are highlighted in Table 2. During postpartum, the main focus of the exercises is to reduce IRD. Until now, there have been no general protocols followed for therapeutic exercises. The approach that therapists choose depends upon the experience and the results obtained by them. Though studies have proven the positive impact of exercises on DRA, the evidence is scarce. The exercise methods concentrate on recruiting the rectus abdominis (RA) and/or transverse abdominis (TrA) muscles. Analyses of these methods are required, since no agreement exists for their effectiveness in the literature. This section provides a summary of the physiotherapeutic exercises prescribed to DRA patients and their outcomes, as reported in the literature.

Sancho et al. conducted an analysis on the impact of exercise on the IRD of postpartum women [40]. This study included 16 postpartum primiparous women during rest and three abdominal strengthening exercises. The exercises included an abdominal crunch, drawing-in, and drawing-in with abdominal crunch. An ultrasound transducer was placed 2 cm above the umbilicus, transversely along the midline of the abdomen. The study revealed significant differences in the IRD between rest and abdominal strengthening exercises. Although there was a notable reduction in IRD, the authors implied that other parameters, such as changes in intra-abdominal pressure, should be examined before prescribing this exercise program. A similar ultrasound study performed by Sancho et al. compared IRD during rest and the three previously mentioned abdominal strengthening exercises. Measurements were performed at two locations, 2 cm above and below the umbilicus. Significant differences were found between rest and abdominal crunch above the umbilicus, and no differences below the umbilicus. Hence the authors concluded that the reduction in IRD was achieved by the abdominal crunch exercise, whereas drawing-in abdominal crunch exercises were ineffective in reducing the IRD [41]. A preliminary case-control study by Pascoal et al [42]. determined the effect of isometric contraction of the abdominal muscles on IRD in postpartum women. The study consisted of two groups of 10 women in each. The experimental group consisted of 10 postpartum women, and the control group consisted of 10 nulliparous women. IRD was measured at 2 cm above the umbilicus using an ultrasound transducer during both rest and while performing abdominal crunch exercises. The authors found that IRD was higher in postpartum women than in nulliparous women, and IRD was found to be lower during an abdominal crunch than at rest.

Mota et al. conducted a longitudinal descriptive exploratory study. This study evaluated the immediate effect induced by drawing-in and abdominal crunch exercises on IRD in 84 primiparous women, through four testing sessions at three locations of the linea alba. The four testing sessions were at 35–41 gestational weeks, 6–8, 12–14, and 24–26 weeks postpartum. The three locations were at 2 cm below the umbilicus, and 2 and 5 cm above the umbilicus. When the patients performed abdominal crunch exercises, there was a significant narrowing of the IRD at all three locations, during all four testing sessions, except at 2 cm below the umbilicus during postpartum week 24–26. When the patients performed the drawing-in exercise, a significant narrowing of the IRD at gestational week 35–41 and widening of the IRD at 6–8, 12–14, and 24–26 postpartum weeks was reported at 2 cm below the umbilicus, as well as a minimally-significant widening of the IRD at 2 and 5 cm above the umbilicus. Therefore, the authors found that abdominal crunch exercises led to the narrowing of IRD in pregnancy and postpartum, whereas the drawing-in exercise led to the widening of IRD [43,44]. A recent transversal experimental study, conducted by Cuña-Carrera et al., studied the immediate effects of three abdominal exercises on IRD. The three abdominal exercises were abdominal crunch, abdominal crunch with transversus abdominis preactivation, and a hypopressive exercise. The IRD was measured just above the umbilicus (U point) and halfway between the U point and the xiphoid (UX point) during rest and performing the three abdominal exercises. With the obtained results, the authors found that the abdominal crunch with transversus abdominis preactivation influenced a significant increase in IRD when compared to rest in UX point, abdominal crunch, and hypopressive exercise in U point [45].

The ultimate aim of the therapeutic exercises in postpartum women with DRA is to reduce the increased IRD. However, Lee et al. presented a distinct notion from the others. They claimed that if the sole purpose of the exercises are only to reduce the IRD, the functional outcome may not be restored. Hence, they proposed rehabilitative exercises that would restore and provide functional and cosmetic benefits. They conducted a study with 26 women diagnosed with DRA and 17 controls. Patients were asked to perform automatic curl-up (Auto-CU) and curl-up with TrA muscle preactivation (TrA-CU). The TrA-CU tenses and pulls the linea alba laterally. An ultrasound transducer was used to measure the IRD immediately above the umbilicus and halfway between this point and the xiphoid. The distortion index was measured as an estimate of the linea alba tension. A comparison in measurements was made at both measurement points, for both groups and during rest, Auto-CU, and TrA-CU; When women with DRA performed Auto-CU, there was a decrease in IRD compared to during rest, but there was an increase in the distortion of the linea alba. When the same women performed TrA-CU, there was a decrease in IRD, but less than with Auto-CU. However, a decrease in distortion of the linea alba was witnessed. Hence, the authors suggested that TrA contraction, which has not been encouraged in rehabilitation, might benefit functional outcomes. They also implied that therapists should not focus solely on the exercises which reduce IRD, especially when such exercises increase the distortion of the linea alba [46,47].

### 4.2. Surgery

Conservative therapy is the initial treatment prescribed for DRA. However, when conservative therapy seems ineffective in severe cases, women are directed towards surgical intervention. Both treatments aim for improvements in the morphological, functional, and quality of life of patients. Surgical treatments for DRA can be classified into different categories, such as open, invasive techniques, laparoscopic, endoscopic, etc. 

#### 4.2.1. Open Approach

In open surgical treatment, the recurrence and complication rates are generally low. Only minor complications exist. Abdominoplasty is the most commonly used technique for DRA. It is performed with or without the plication technique. Abdominoplasty is used to treat excess skin, whereas plication improves aesthetics and function. The incision may be transverse suprapubic, midline supraumbilical, left suprapubic, and midline ventral. In cases where diastasis recti are concomitant with a hernia, abdominoplasty is performed along with a peri-umbilical incision. Simple plication of the linea alba is performed in cases of mild to moderate diastasis recti. This procedure can be performed in an open, laparoscopic, or hybrid approach, with or without the use of mesh. The suture layer can be single or double in an interrupted or continuous manner. Retro rectus repair reinforcement with sublay mesh, based on the Rives-Stoppa principles, is performed in the cases of moderate to severe diastasis recti [48,49].

Sutures are of two types, absorbable and non-absorbable. The non-absorbable and absorbable sutures used to correct DRA are nylon and polydioxanone, respectively. Nahas et al. performed a study to verify the efficacy of the plication of the anterior rectus sheath by comparing the use of both sutures. This study revealed that the correction of DRA using both nylon and polydioxanone is achievable. A long-term follow-up of the procedure correcting the rectus diastasis using non-absorbable sutures was performed by Nahas et al. This study revealed that there was no recurrence in any of the patients. Hence the authors concluded that plication of the anterior rectus sheath with non-absorbable sutures seemed to be a long-lasting procedure for the correction of DRA. Nahas et al. conducted another long-term follow-up for the correction of rectus diastasis, but by using an absorbable polydioxanone suture for the plication of the anterior rectus sheath. This study also revealed that there was no recurrence of DRA in any of the patients. Hence the authors concluded that plication of the anterior rectus sheath with nonabsorbable sutures also seems to be a long-lasting procedure for the correction of DRA [50,51,52]. Mestak et al. conducted a case-control study in 51 patients to evaluate the outcomes of DRA correction using absorbable sutures. The authors found no statistically significant differences in IRD between the study and the control group. Hence the rectus sheath plication using absorbable sutures in patients with DRA was found to be a reliable method that tends to maintain the long-term stability of the abdominal wall [53].

In a few cases, DRA presents with an umbilical hernia. In such cases, abdominoplasty with a periumbilical incision is a preferred method of treatment. This procedure results in an umbilical incision or an inverted T scar. A retrospective cohort study conducted by J. Kulhanek et al. involved a limited scar abdominoplasty without a periumbilical incision. This procedure is different from the traditional abdominoplasty. This method was reported to be an excellent method, since it results in small and hidden scars, and is beneficial for young and slim women [18]. The changes in the length of the musculoaponeurotic layer after DRA repair using triangular mattress sutures were assessed by Veríssimo et al. The authors achieved a vertical shortening of the musculoaponeurotic layer immediately after the procedure, and in the long-term, when performed using triangular mattress sutures [54]. Mabrouk et al. evaluated the long-term efficiency of two different methods used for the plication of the anterior rectus sheath during abdominoplasty. The patients were divided into two groups in a random manner. Group 1 consisted of patients which underwent the rectus abdominis “myofascial release”. Group 2 consisted of patients which underwent conventional midline plication of the external oblique aponeurosis. In both operative groups, there was a significant reduction in the mean IRD before surgery and twelve months postoperatively. There was no recurrence in either group, except one in the first group, and two in the second group [55]. A study conducted by Gama et al. compared the efficacy and time required to repair DRA using different plication techniques. The authors found that the plication of the anterior rectus sheath in a single-layer using a continuous suture showed to be an efficient and rapid technique for the repair of DRA [56]. Köhler et al. performed a minimal invasive linea alba reconstruction (MILAR) with the supra-aponeurotic placement of a fully absorbable synthetic mesh in patients. It allows the formation of a new linea alba with the augmentation of autologous tissue consisting of plicated anterior rectus sheaths. The supra-aponeurotic placement of a fully absorbable synthetic mesh allows for the elimination of potential long-term mesh-associated complications [57]. A prospective cohort study by Olsson et al. determined the effect of surgical repair of DRA on QoL, abdominal trunk function, and urinary incontinence in postpartum women with training-resistant symptoms and trunk instability. This surgical technique resulted in significant improvements in abdominal trunk function, urinary incontinence, and QoL [58].

A randomized controlled clinical trial (RCT) conducted by Emmanuelsson et al. evaluated the early complications and patient satisfaction, by comparing the retromuscular inset of a lightweight polypropylene mesh with dual closure of the anterior rectus fascia. This RCT was performed using sixty-four patients out of which fifty-seven patients were allocated for surgery. Out of the fifty-seven patients, twenty-nine were reconstructed with mesh, and twenty-eight patients underwent repair with slowly-absorbed 2-0 PDO barbed self-anchoring sutures. After a three-month follow-up no substantial changes were observed for the two techniques performed. Both the techniques may be equally reliable, although patients who underwent the mesh technique experienced better muscular strength improvement. The authors conducted a long-term follow-up for the same study to compare long-term recurrence after surgery, abdominal muscle strength, pain, and quality of life. During the follow-up phase, a few patients reported discomfort and diffuse pain. Most of the patients in both groups did not express a difference in well-being and the same outcome for core stability. Many of the patients in both groups were satisfied with the functional outcome, but only a few in the aesthetic outcome. The implantation of retromuscular mesh requires more extensive surgery than the double-row suture repair, thus having a higher potential risk for complications. Therefore, the authors recommended the double-row self-retaining suture technique, since this group of patients had less pain and improvements in QoL [59].

Another RCT conducted by the same authors evaluated the risk of recurrence using two operative techniques for DRA. They also compared the pain, abdominal muscle strength, and quality of life of the group who underwent surgery with the control group, who received physical training only. Out of the eighty-six patients enrolled, twenty-nine were allocated retromuscular polypropylene mesh, twenty-seven patients, double-row plication with Quill technology, and thirty-two patients, a three-month physical training program. Abdominal muscle strength was assessed using the Biodex System, and pain was evaluated using the ventral hernia pain questionnaire and quality of life survey. There was one early recurrence in the Quill group. Two patients were found with an encapsulated seroma in the mesh group, and three patients in the Quill group. At one-year follow-up, there were significant improvements in perceived pain and quality of life, showing no differences between the two groups. In all groups, there were significant muscular improvements. In both the operative groups, there were similar improvements with patients who perceived gain in muscular strength. This improvement was higher in operative groups than in training groups, where the patients still experienced bodily pain during follow-up. Neither technique showed a difference in terms of abdominal wall stability, and both had a similar complication rate [60]. Çintesun et al. investigated the relationship between the re-approximation of the rectus muscles during cesarean section and the severity of DRA in the first operative month. The authors found that the re-approximation of rectus muscles does not influence the prevention of diastasis recti [61].

#### 4.2.2. Laparoscopic Approach

Laparoscopy provides a minimally invasive form of surgery. The short-term results provided by laparoscopic surgeries have increased interest among surgeons. This technique has reported favorable outcomes and low recurrence rates without using a prosthesis. Indications for laparoscopic surgery are believed to be cosmesis and the functional return of abdominal muscles. Overall, this approach is found to be effective and beneficial for the correction of DRA.

Palanivelu et al. described their method of plication using laparoscopy. The ‘Venetian blinds’ technique, combined with prosthetic mesh reinforcement, was performed on patients. A CT scan was performed to measure the IRD preoperatively and postoperatively. After 6–48 months of follow-up, the recurrence rate was zero percent. The ‘Venetian blinds’ technique is an effective form of plication, providing additional support, and thereby reducing the risk of seroma [62]. A case with a successful outcome from laparoscopic plication of the linea alba, without using mesh, was presented by Siddiky et al. A subject diagnosed with DRA was operated on and the reduction in IRD was recorded during the operation. The benefits reported by the authors were improved recovery time, reduced pain, and wound negation. Since this procedure is performed without the use of mesh, it avoids the risks involved with the insertion of foreign material into the body [63]. A new technique called subcutaneous onlay laparoscopic approach (SCOLA) was described by Claus et al. for the correction of ventral hernias combined with DRA plication. This technique was performed on patients indicated by a primary abdominal or incisional hernia with concomitant DRA. Out of the reported patients, four were operated on with the help of a robotic platform, and the remaining subjects were operated on by conventional laparoscopy. Mesh was not used in three cases. No intraoperative complications were recorded. Postoperative complications occurred in fifteen patients. Recurrence was only reported after eight months of follow-up [20].

Fiori et al. performed a retrospective study to demonstrate an overall approach to DRA leading to good functional and morphological outcomes and quality of life. This study involved three different surgical procedures, namely laparoabdominoplasty, laparominiabdominoplasty and an endoscopic technique. A study was conducted with ninety-four patients to assess the interoperative complications of the above-mentioned surgical procedures. Out of the reported subjects, thirty-nine underwent laparoabdominoplasty, twenty-nine underwent laparominiabdominoplasty, and twenty-six had endoscopic procedures. No intraoperative complications or recurrences were reported. Major complications occurred in three cases only after surgery [16]. A recent study proposed by Manetti et al. described a technique by modifying the Costa’s technique. It combines Rives-Stoppa principles and minimally invasive access to plicate the posterior rectus sheaths using a surgical stapler. A clinical questionnaire was used to analyze the outcomes of the treatment. After two months from surgery, follow-up began. No major complications, readmissions, or postoperative infections were reported. Two recurrences were registered after a timeframe of six months. This technique has also achieved promising results, although a long-term follow-up is necessary [49].

#### 4.2.3. Endoscopic Approach

The first abdominoplasty using endoscopy was performed in 1991. Endoscopic procedures are minimally invasive. Minimally invasive techniques have obtained preference over open techniques. They possess a decreased morbidity and lower recurrence rates. Failure of the procedure may be converted into an open approach at any time. Hence surgeons performing the endoscopic procedure should also be familiar with the open procedure.

An earlier study by Core et al. in 1995 performed endoscopic abdominoplasty along with the repair of DRA. This technique was performed on two women. After three months of follow-up, there was a complete absence of transverse scarring in the pubic region of both patients. It might also serve as a standard plastic surgery tool [64]. Bellido Luque et al. performed a prospective cohort study to prospectively evaluate the feasibility and duration of the plication of both aponeuroses using a totally endoscopic approach. This approach is used in patients having DRA associated with midline hernias. This surgery was performed on twenty-one patients. An ultrasound scan was used to measure the IRD. Patients were followed up with a mean period of twenty months. At one-month follow-up, there was a significant reduction in average IRD before and after surgery, in all three locations, whereas no significant differences were seen in the first to second year. Seroma was reported as the main complication. There was a significant improvement in the cosmetic outcome in the first operative year. After surgery, significant improvements were seen in back pain. Therefore, the authors concluded that this method is feasible and reproducible [65].

Endoscopic-assisted linea alba reconstruction plus mesh augmentation (ELAR plus) is a hybrid technique used by Köckerling et al. for the reconstruction of the linea alba and mesh augmentation. This surgery was performed in patients with symptomatic epigastric and/or umbilical hernia concomitant with DRA. Out of the reported patients, only two patients had impaired umbilical wound healing, and one had a seroma. All of these complications were treated conservatively. Three patients complained of intermittent pain on exertion, and two took painkillers as and when required. The authors concluded that ELAR plus is a novel, minimally invasive technique, and permits the restoration of the normal anatomy of the abdominal wall [66]. Gandhi et al. described a new technique, endoscopic pre-aponeurotic repair (EPAR), for the correction of ventral hernias combined with the plication of DRA, with meshplasty. The authors concluded that this technique was a safe and effective alternative for treating patients with ventral hernias along with DRA [19]. A new endo-laparoscopic technique for midline reconstruction, for subjects with diastasis recti, was analyzed by Carrara et al. This technique was performed in 110 consecutive patients as a prospective observational study. The patients were subjected to the endo-laparoscopic reconstruction of the abdominal wall using linear staplers. Follow-up was performed after surgery. At six-months follow-up, a mean IRD of 6.7 mm was reported. During the mean follow-up of 14 months, the morbidity rate was recorded as 9.1%. Since this method has shown significant improvements in patients’ quality of life, reduction of abdominal wall pain, low back pain, bulging, and stress urinary incontinence, the authors concluded this technique to be a safe, feasible, and effective alternative method. They also recommend long-term follow-up, since the short- and mid-term results were encouraging [67].

## 5. Diagnostic Techniques for the Rehabilitation of DRA

With the application of engineering principles, a rehabilitative procedure offers better solutions to differently abled individuals. It bridges the gap between the disabilities of an individual and their interaction with the environment. Currently, various advances have been made to provide optimized solutions in the field of rehabilitation engineering. Rehabilitation procedures in physiotherapy have aided in the effective recovery process of patients. This tool has also made treatment protocols much easier. This section gives an overview of the role of Electromyography (EMG) and Ultrasound imaging as diagnostic technologies for abdominal muscles in therapeutic exercises.

### 5.1. Electromyography

Electromyography is a technique that detects the electrical activity of a muscle during contraction. EMG can be measured in two ways, by using surface or needle electrodes. The surface electrode is noninvasive and the most commonly used. The signal recorded by the surface electrode is known as the surface EMG (sEMG) signal. The applications of sEMG are medical, rehabilitation, ergonomics, and sports science. The application of EMG in exercise therapy allows for the analysis of movements performed by patients. EMG studies for abdominal muscles in exercise therapy have been recorded in the literature. These studies have investigated muscle activation during various movements performed in different positions. Electromyography (EMG) facilitates the assessment of the effectiveness of each exercise and allows the recording of muscle activity produced during the movement. EMG is considered a gold standard for recording muscle activity. The EMG signal provides valuable information relative to the muscles activated and the movements performed. The higher the electrical activity of the muscles, the larger the number of muscle fibers recruited, thereby improving muscle strength. An electromyographic investigation on the abdominal exercises conducted by Robinson et al. concluded that lower and upper RA muscles are predominantly activated during the abdominal exercises [68]. Hwang et al. utilized EMG to investigate abdominal muscle activity during an abdominal drawing-in maneuver combined with irradiation variations. This study implies that irradiation is helpful for increasing the activities of all the abdominal muscles, but has a confined effect on the TrA muscle alone [69]. Traditional and nontraditional abdominal exercises analysis using electromyography was carried out by Escamilla to test their effectiveness. This study investigated 12 different exercises activating the abdominal muscles by movements, such as trunk flexion, hips flexion with posterior pelvis rotation, a combination of trunk flexion and hip flexion with posterior pelvis rotation, and resting trunk extension. The EMG analysis of the exercises was carried out to determine the effectiveness of each exercise in activating the abdominal muscles [70]. Another similar study was conducted on five different exercises activating the abdominal muscles. 

### 5.2. Ultrasound Imaging

Ultrasound imaging (USI) is a noninvasive imaging technique used to visualize internal organs of the body. It uses high frequency sound waves to produce images of the internal organs. USI can also be used to visualize functions of the internal organs, muscle movements, and blood velocity. USI is currently used extensively in rehabilitation. Its application in physiotherapy has led to various findings in musculoskeletal studies. Muscle re-education, training, size, and thickness measurement are some of the potential applications of USI in rehabilitation. USI is found to be highly reliable for the assessment of abdominal muscle during the drawing-in exercise when performed by novice assessors, although, there were a few inconsistencies in the measurement’s factors. For a few measurements, the assessors also displayed varied reliability. Therefore, the assessors require proper training before using ultrasound in rehabilitation [71]. A cross-sectional study design by Teyhen et al. utilized USI to characterize changes in the muscle thickness of TrA and internal oblique muscles during trunk-strengthening exercises [72]. USI was also employed to measure the abdominal wall changes during lumbar stabilization exercises in patients with lower back pain [73]. Aboufazeli et al. conducted a study to investigate the intraday and interday reliability of abdominal muscle thickness measurement during abdominal hollowing and bracing maneuvers using USI. They recommended USI as a reliable method to determine the thickness of TrA and IO muscles, and to some extent, external oblique muscles [74]. Pirri et al. presented a study to assess the inter-rater reliability and variability of ultrasound measurements for abdominal muscles. This study inferred that the USI serves to be a noninvasive, reliable and cost-effective modality to analyze abdominal muscles [75]. Fan et al. presented a study to analyze the effects of vaginal delivery and cesarean section (C-Section) on abdominal muscles and fasciae [76]. It was reported that women having a cesarean section showed significant alterations in both muscle thickness and abdominal fasciae; but for women with vaginal deliveries, changes were observed only in muscles. 

## 6. Discussion

This work summarizes the causes of DRA, its diagnosis, treatment, and the role of abdominal muscle therapeutic exercise in better rehabilitation. Only a few reviews for DRA exist, and the scope of these works are limited to the analysis of treatment procedures [77]. A detailed analysis of DRA is also scarce in current literature. However, the thematic integration of the literature has produced numerous valuable insights, and provides a number of suggestions for further research.

### 6.1. Impact of Exercises

Conservative treatment is considered as the first-line treatment for DRA, and it still lacks proper investigation. Studies on therapeutic exercise provide varied results. The exercises suggested by therapists target abdominal muscles and focus on reducing the IRD. The muscles targeted are RA and TrA. These exercises do not affect lateral abdominal muscles. Limited information was found in the literature regarding which exercises provide promising outcomes as the result of the studies. While many therapists propose exercises to reduce IRD which enhance cosmetic outcomes, Lee et al. offers a different opinion on how to restore the functional abilities of the linea alba. According to the authors, it is more important to restore the tension of the linea alba than to reduce the IRD. Since different authors approach the problem differently, it is necessary to conduct further research to find an optimal strategy which enhances both functional and cosmetic outcomes.

### 6.2. Need for a Biofeedback Device

Some patients might lack the ability to precisely follow the instructions given by therapists. Hence, there are more chances for patients to activate a different muscle than the targeted one. This might lead to a negative influence on the outcomes of the exercise program. This implies the need to develop a device which provides biofeedback to the patient while constantly performing the exercises. A biofeedback device might provide valuable audio or visual information to the patients about the physical activity. It would allow patients to engage in performing the exercises through continuous training, which may allow patients to develop fundamental abilities to perform the exercises well. This device could only be used to augment the training provided by therapists, and would not replace therapists by any means.

### 6.3. Scope of Automation

As far as the methods to measure IRD are concerned, the techniques mentioned in the literature are manually performed by therapists using fingers, calipers, or ultrasound imaging. The ultrasound imaging technique, considered the gold standard to measure IRD, requires an experienced person to examine, and is quite expensive for patients to afford in developing countries. Hence the daily use of ultrasound imaging is limited to clinical practice. Therefore, developing an automatic assessment of IRD would be extremely beneficial for therapists, as well as patients. The system developed must be highly reliable, valid, and responsive. It must allow both the patients and therapists to obtain the data from time to time, thereby supporting the continuous monitoring of the IRD. The application of technology in physiotherapy has been increasing greatly. Hence, developing a technology which would provide an automatic assessment of the rehabilitative exercises in DRA patients would be profitable for the therapists. It might allow physiotherapists to measure patient movement, estimate the recovery process, and control and plan exercise protocols for patients.

## 7. Conclusions

DRA is a significant problem encountered by women after pregnancy. This work discusses the treatment options for DRA and highlights the importance of technology in the betterment of treatment. In recent years, minimally invasive surgery has been developed to reduce IRD. However, it is not applicable in all cases. Exercise therapy is encouraged for women, even during the pregnancy period. Although the exercise protocol for DRA needs to be properly standardized, various studies on exercise therapy for DRA patients have revealed significant results. Advancements in technology have made rehabilitation procedures easier to evaluate and have made it easier to prescribe corrective measures. The assessment of exercises using rehabilitation technology allows therapists to monitor the performance and progress of patients. In DRA exercise therapy, most of the exercises concentrate on the abdominal muscles. Electromyography and Ultrasound imaging are valuable tools in the assessment of abdominal muscles during exercises. Hence, further experimental investigations are recommended to utilize these technologies for DRA rehabilitation, to further assess the physiology of abdominal muscles during exercise. 

## Figures and Tables

**Table 1:** Exclusion and Inclusion Criteria.

Factor	Inclusion	Exclusion
Research outcome	Studies involving DRA in postnatal women. Studies dealing with the effect and outcomes of surgical procedures for treating DRA. Studies dealing with ultrasound imaging and abdominal muscles related to vaginal delivery and cesarean section	Studies that only considered healthy controls.
Study design and Methodology	Studies that employ sensor for assessment of abdominal exercises.	Statistical methods for assessment. Biochemical research studies on the properties of the abdominal muscles.

**Table 2:** Details of abdominal exercises prescribed.

S.No	Name of the Exercise	Description
1.	Sit-Up	The exercise is performed by the subject lying with arms straight, knees bent and feet in a flat position. The abdominal muscles are then engaged, while a slight lift of the head, neck and shoulders is performed. The position is held for 10 s and then a return to the starting position.
2.	Curl-up	The exercise is performed by the subject lying with arms straight, knees bent and feet in a flat position. The abdominal muscles are then engaged, while a major lift of the head, neck and shoulders is performed. The position is held for 10 s and then a return to the starting position.
3.	TA Sit-up	The transverse abdominis muscle contraction is done along with sit-up.
4.	TA Curl-up	Transverse abdominis muscle contraction is done along with curl-up.
5.	TAPFM Curl-up	The transverse abdominis muscle and pelvic floor muscle contraction is done along with curl-up.
6.	TASLR	The transverse abdominis muscle contraction is done along with straight leg raise. In the straight leg raise, the subject lies flat initially. One leg is raised above and the position held for 10 s and then relax back to the starting position.
7.	Reverse Curl-up	In a reverse curl up, the subject initially lies flat. Then both legs are curled up together with knees towards chest. Hold the position for 10 s and then relax back to the starting position.
